# Cross-sectional study of the neural ossification centers of vertebrae C1–S5 in the human fetus

**DOI:** 10.1007/s00276-013-1093-5

**Published:** 2013-02-28

**Authors:** Michał Szpinda, Mariusz Baumgart, Anna Szpinda, Alina Woźniak, Celestyna Mila-Kierzenkowska

**Affiliations:** 1 Department of Normal Anatomy, The Ludwik Rydygier Collegium Medicum in Bydgoszcz, The Nicolaus Copernicus University in Toruń, Karłowicza 24 Street, 85-092 Bydgoszcz, Poland; 2 Department of Medical Biology, The Ludwik Rydygier Collegium Medicum in Bydgoszcz, The Nicolaus Copernicus University in Toruń, Karłowicza 24 Street, 85-092 Bydgoszcz, Poland

**Keywords:** Spine, Neural ossification center, Dimensions, CT examination, Digital-image analysis, Spina, Bifida, Human fetus

## Abstract

**Purpose:**

An understanding of the normal evolution of the spine is of great relevance in the prenatal detection of spinal abnormalities. This study was carried out to estimate the length, width, cross-sectional area and volume of the neural ossification centers of vertebrae C1–S5 in the human fetus.

**Materials and methods:**

Using the methods of CT (Biograph mCT), digital-image analysis (Osirix 3.9) and statistics (the one-way ANOVA test for paired data, the Kolmogorov–Smirnov test, Levene’s test, Student’s *t* test, the one-way ANOVA test for unpaired data with post hoc RIR Tukey comparisons) the size for the neural ossification centers throughout the spine in 55 spontaneously aborted human fetuses (27 males, 28 females) at ages of 17–30 weeks was studied.

**Results:**

The neural ossification centers were visualized in the whole pre-sacral spine, in 74.5 % for S1, in 61.8 % for S2, in 52.7 % for S3, and in 12.7 % for S4. Neither male–female nor right–left significant differences in the size of neural ossification centers were found. The neural ossification centers were the longest within the cervical spine. The maximum values referred to the axis on the right, and to C5 vertebra on the left. There was a gradual decrease in length for the neural ossification centers of T1–S4 vertebrae. The neural ossification centers were the widest within the proximal thoracic spine and narrowed bi-directionally. The growth dynamics for CSA of neural ossification centers were found to parallel that of volume. The largest CSAs and volumes of neural ossification centers were found in the C3 vertebra, and decreased in the distal direction.

**Conclusions:**

The neural ossification centers show neither male–female nor right–left differences. The neural ossification centers are characterized by the maximum length for C2–C6 vertebrae, the maximum width for the proximal thoracic spine, and both the maximum cross-sectional area and volume for C3 vertebra. There is a sharp decrease in size of the neural ossification centers along the sacral spine. A decreasing sequence of values for neural ossification centers along the spine from cervical to sacral appears to parallel the same direction of the timing of ossification. The quantitative growth of the neural ossification centers is of potential relevance in the prenatal diagnosis and monitoring of achondrogenesis, caudal regression syndrome, diastematomyelia and spina bifida.

## Introduction

Three-dimensional ultrasonography plays a critical role in assessing and monitoring most fetal structures [8, 23–25], including the fetal spine after the 12th week of gestation [10, 11, 21, 22, 26, 28, 29]. Ossification of every vertebra starts with three primary centers, one body ossification center, and a pair of neural ossification centers [1–4, 6, 18, 20], which independently of each other evolve in the spine in a definite regional sequence [2]. The vertebral bodies begin to ossify in the distal thoracic-proximal lumbar spine and simultaneously progress both cranially and caudally [2, 22]. The topographical sequence of neural ossification centers is somewhat ambiguous with the three possible spinal origins: at the same time in the thoracolumbar, cervico-thoracic and proximal cervical segments [5], or in the mid-thoracic segment [19], or in the proximal cervical segment [3]. The detailed knowledge on neural ossification centers appears to be an immense prerequisite for both the prenatal detection and exclusion of achondrogenesis, caudal regression syndrome, diastematomyelia [17, 26, 28], and spina bifida [11, 15]. Besides, delayed ossification centers are typical of osteochondrodysplasias [9, 28] and hypophosphatasia [31].

Szpinda et al. [22] have recently published a cross-sectional study of the ossification center of the C1–S5 vertebral bodies. Except for the precise morphometric study on the three ossification centers of C4 and L3 vertebrae in the human fetus [4, 25], so far there has been incomplete information concerning quantitative analysis of neural ossification centers. To examine this question specifically, in this study we aimed the following.

- To determine the length, width, cross-sectional area and volume for the neural ossification centers of vertebrae C1–S5.
- To examine the impact of sex on the values obtained.
- To display graphically the growth of every parameter for the individual C1–S5 vertebrae in different age-groups and for the whole sample.

## Materials and methods

**Fig. 1:**
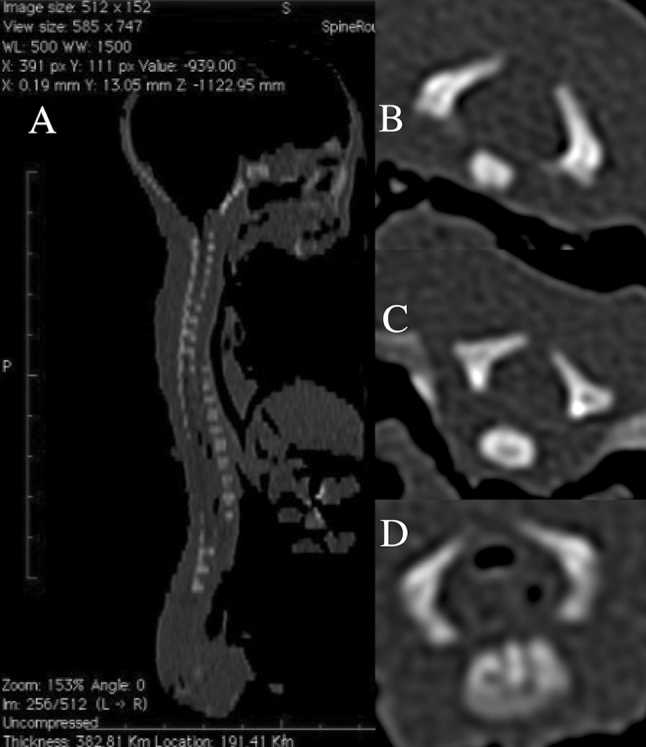
CT of a female fetus aged 24 weeks (in the sagittal projection) recorded in DICOM formats (**a**) with spinal ossification centers (in the transverse projection) of C4 (**b**), T6 (**c**), and L3 (**d**) vertebrae, being assessed by Osirix 3.9

This examination included 55 human fetuses (27 males, 28 females) of Caucasian racial origin at ages of 17–30 weeks (Table 1), which had come from spontaneous abortions or stillbirths in the years 1989–2001 as a result of placental insufficiency. Gestational ages were calculated by the crown-rump length [12]. The use of the fetuses for research was approved by the University Research Ethics Committee (KB 275/2011). All fetuses were free from conspicuous external malformations. After having been immersed in 10 % neutral buffered formalin, the fetuses underwent CT examinations with the reconstructed slice width option of 0.4 mm. As a result, 128 slices were simultaneously acquired by Biograph mCT (Siemens). No bone showed evidence of mal-development. The scans obtained were stored in DICOM formats (Fig. 1a) with possibility both to compute three-dimensional reconstructions and to analyze chosen objects. The gray scale of obtained CT images in Hounsfield units ranged relatively widely, from −275 to −134 for a minimum, and from +1,165 to +1,558 for a maximum. As a consequence, the window width (WW) ranged from 1,404 to 1,692, and the window level (WL) varied from +463 to +712. As the next step, DICOM formats were evaluated by digital image analysis of Osirix 3.9 (Fig. 1b), which semi-automatically estimated linear (length, width), two-dimensional (cross-sectional area), and three-dimensional (volume) parameters of the neural ossification centers of vertebrae C1–S5 (Fig. 1c, d). The contouring procedure for each body ossification center was outlined with a cursor and stored.

**Table 1:** Distribution of the fetuses studied

Gestational age (weeks)	Crown-rump length (mm)				Number	Sex	
	Mean	SD	min	max		Male	Female
17	115.00		115.00	115.00	1	0	1
18	133.33	5.77	130.00	140.00	3	1	2
19	149.50	3.82	143.00	154.00	8	3	5
20	161.00	2.71	159.00	165.00	4	2	2
21	174.75	2.87	171.00	178.00	4	3	1
22	185.00	1.41	183.00	186.00	4	1	3
23	197.60	2.61	195.00	202.00	5	2	3
24	208.67	3.81	204.00	213.00	9	5	4
25	214.00		214.00	214.00	1	0	1
26	229.00	5.66	225.00	233.00	2	1	1
27	239.17	3.75	235.00	241.00	6	6	0
28	249.50	0.71	249.00	250.00	2	0	2
29	253.00	0.00	253.00	253.00	2	0	2
30	263.25	1.26	262.00	265.00	4	3	1
Total					55	27	28

**Fig. 2:**
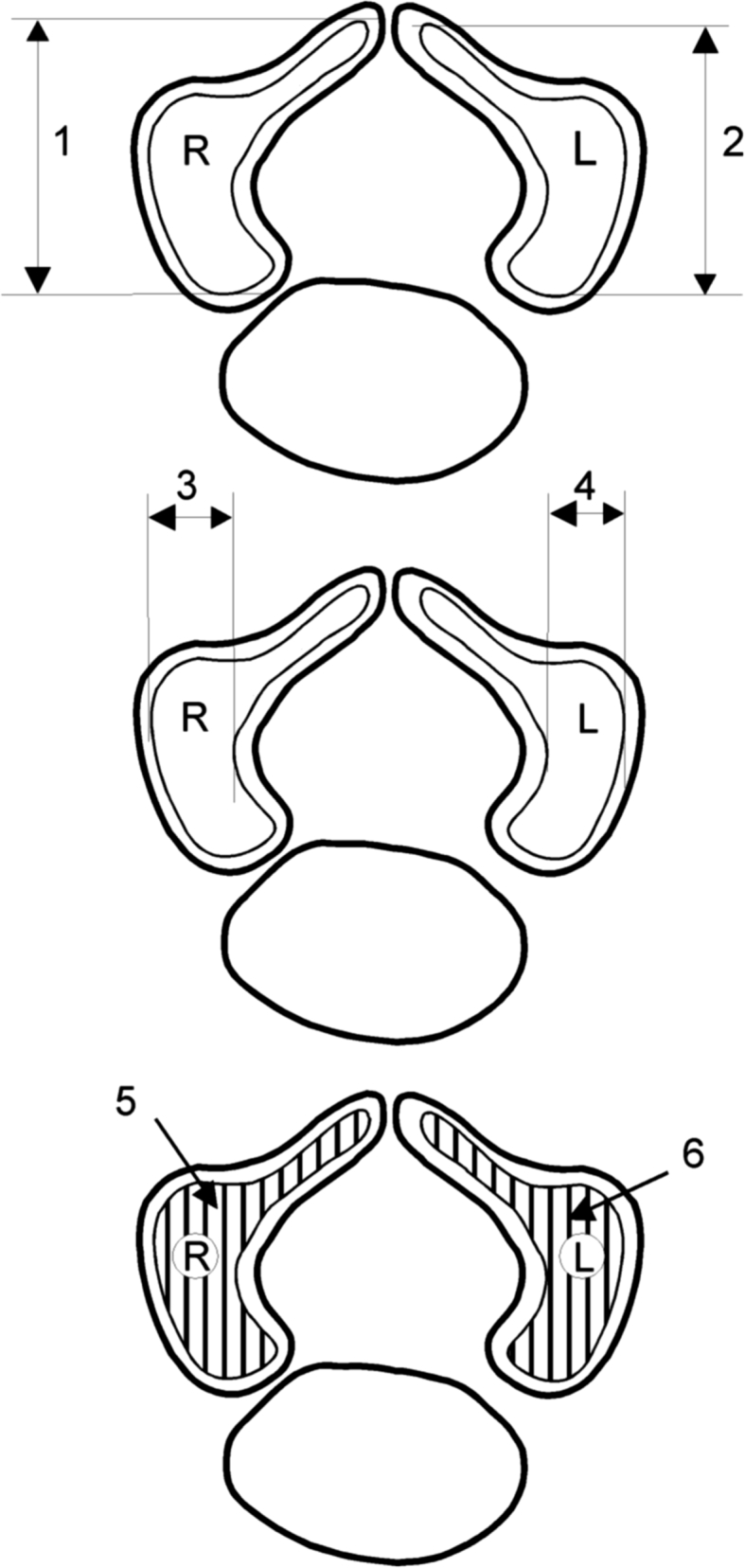
Diagram showing different measurements (apart from volumes) of the neural ossification centers of vertebrae C1–S5: length of the right *1* and left *2* neural ossification centers, width of the right *3* and left *4* neural ossification centers, cross-sectional area of the right *5* and left *6* neural ossification centers; *R* right neural ossification center, *L* left neural ossification center

For every fetus, the following eight parameters of the neural ossification centers of C1–S5 vertebrae were evaluated (Fig. 2):

- 1, 2. right and left lengths (in mm), corresponding to the distance between the anterior and posterior borderlines of the neural ossification center (in transverse projection),
- 3, 4. right and left widths (in mm), corresponding to the distance between the left and right borderlines of the neural ossification center (in transverse projection),
- 5, 6. right and left cross-sectional areas (in mm^2^), traced around the neural ossification center(in transverse projection),
- 7, 8. right and left volumes (in mm^3^).

So as to minimize measurement and observer bias as much as possible, all measurements were done by one researcher (M.B). Each measurement was repeated three times under the same conditions but at different times, and the mean of the three was finally accepted. The 6,380 obtained results were subjected to statistical analysis. The differences between the repeated measurements, as the intra-observer variation, were evaluated by the one-way ANOVA test for paired data. The data obtained were checked for normality of distribution using the Kolmogorov–Smirnov test and homogeneity of variance with the use of Levene’s test. For statistics the fetuses were separated in a following way: group I (17–19 weeks) 12 specimens, group II (20–23 weeks) 17 specimens, group III (24–27 weeks) 18 specimens, and group IV (28–30 weeks) 8 specimens. At first, Student’s *t* test was used to examine whether or not sex influenced the values obtained. To examine sex differences, we checked possible differences between the following four age groups: 17–19, 20–23, 24–27, and 28–30 weeks of gestation. Afterwards, we tested possible sex differences for the whole sample, irrespective of fetal ages. To check whether or not variables changed significantly with age, the one-way ANOVA test for unpaired data, and then post hoc RIR Tukey comparisons were used for the four aforementioned age groups. By plotting the numerical data of every parameter of the neural ossification center versus the associated vertebra we obtained curves for their relative growth.

## Results

In the examined material all the neural ossification centers of the pre-sacral spine were visualized. This stood out in stark contrast when compared to the neural ossification centers of the sacral spine, being visible in 41 (74.5 %) fetuses for S1, 34 (61.8 %) fetuses for S2, 29 (52.7 %) fetuses for S3, 7 (12.7 %) fetuses for S4, and in no fetus for S5.

**Fig. 3:**
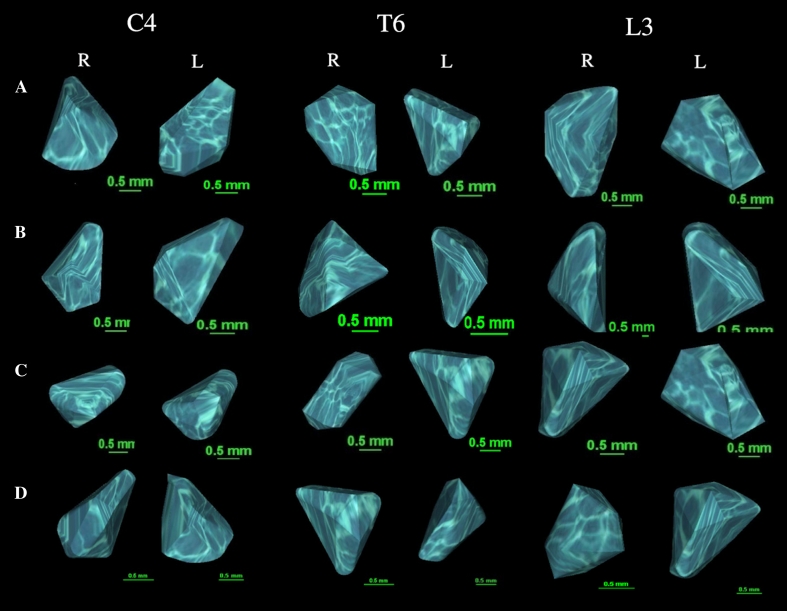
Right (R) and left (L) neural ossification centers of C4, T6 and L3 vertebrae in fetuses aged 18 weeks (**a**), 21 weeks (**b**), 25 weeks (**c**), and 29 weeks (**d**)

No statistically significant differences (*P* > 0.05) were found in the evaluation of intra-observer reproducibility of the measurements of neural ossification centers of C1–S5 vertebrae. The morphometric values obtained were characterized by normality of distribution and homogeneity of variance. Since no significant difference was observed in values of the parameters studied according to sex, no attempt was made to further separate the results obtained according to males and females. By contrast, advancing gestational age was characterized by a statistically significant (*P* = 0.01) increase in values of all measurements. Figure 3 presents right (R) and left (L) neural ossification centers for C4, T6 and L3 vertebrae in fetuses aged 18 weeks (A), 21 weeks (B), 25 weeks (C), and 29 weeks (D).

**Fig. 4:**
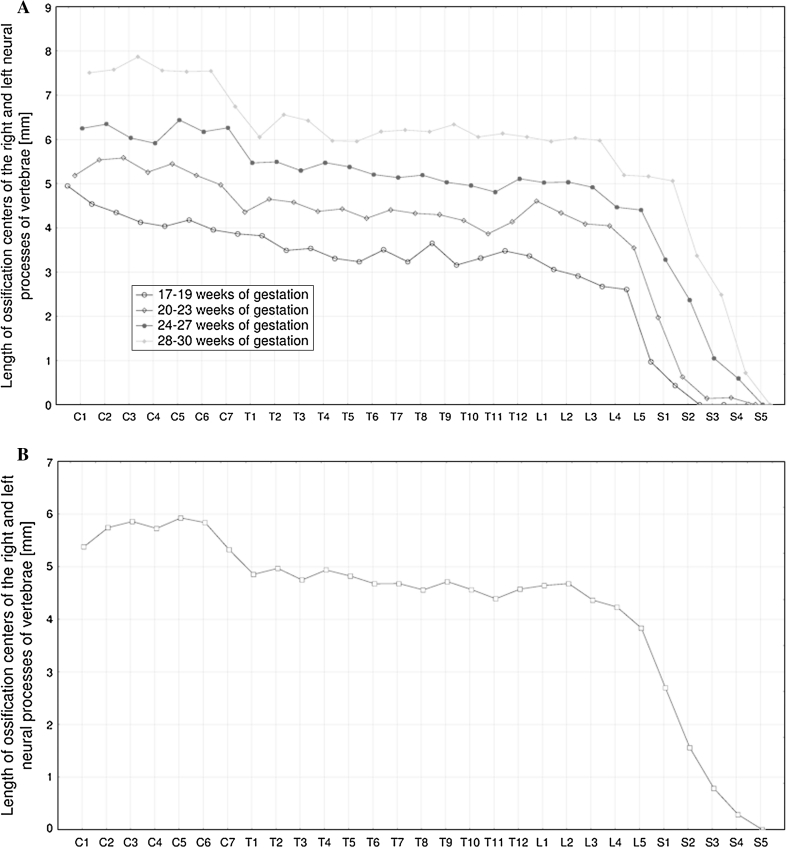
Mean lengths of neural ossification centers of the C1–S5 vertebrae in fetuses aged: 17–19, 20–23, 24–27, and 28–30 weeks of gestation (**a**), and for all fetuses (**b**)

**Fig. 5:**
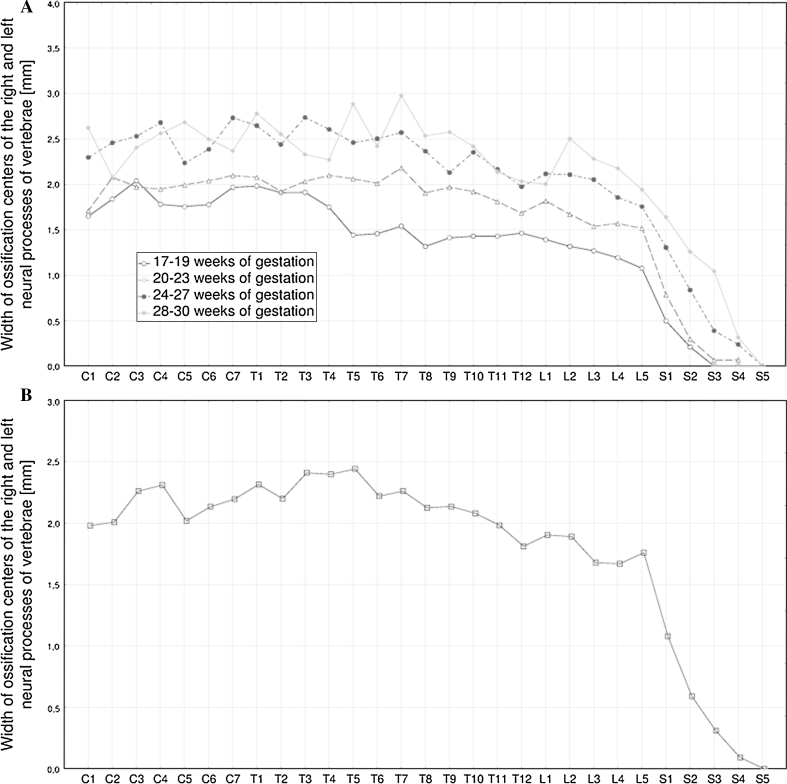
Mean widths of neural ossification centers of the C1–S5 vertebrae in fetuses aged: 17–19, 20–23, 24–27, and 28–30 weeks of gestation (**a**), and for all fetuses (**b**)

**Fig. 6:**
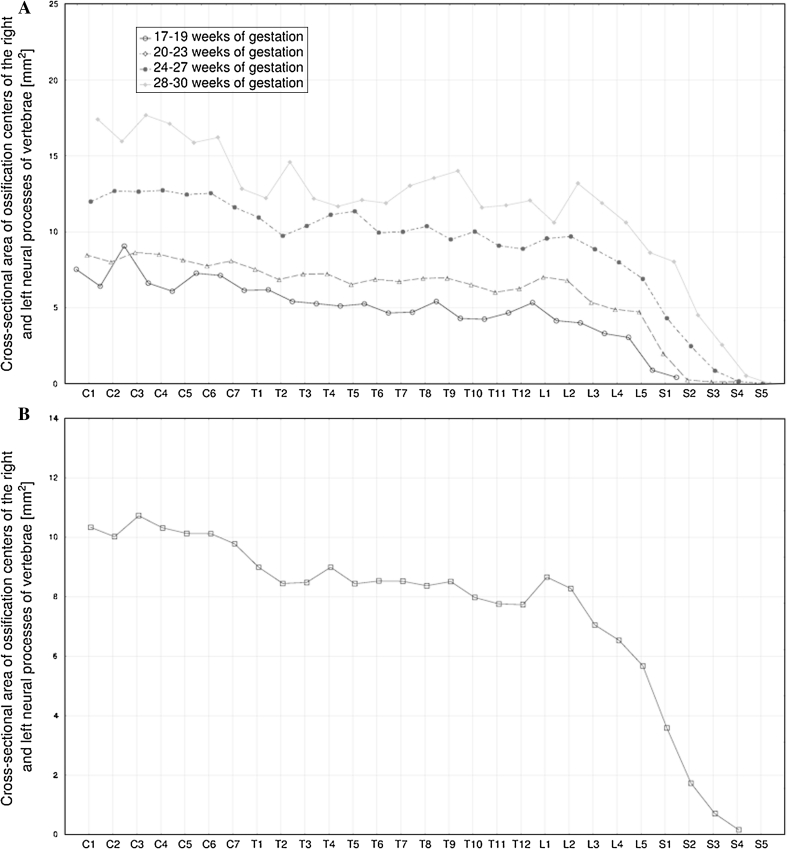
Mean cross-sectional areas of neural ossification centers of the C1–S5 vertebrae in fetuses aged: 17–19, 20–23, 24–27, and 28–30 weeks of gestation (**a**), and for all fetuses (**b**)

**Fig. 7:**
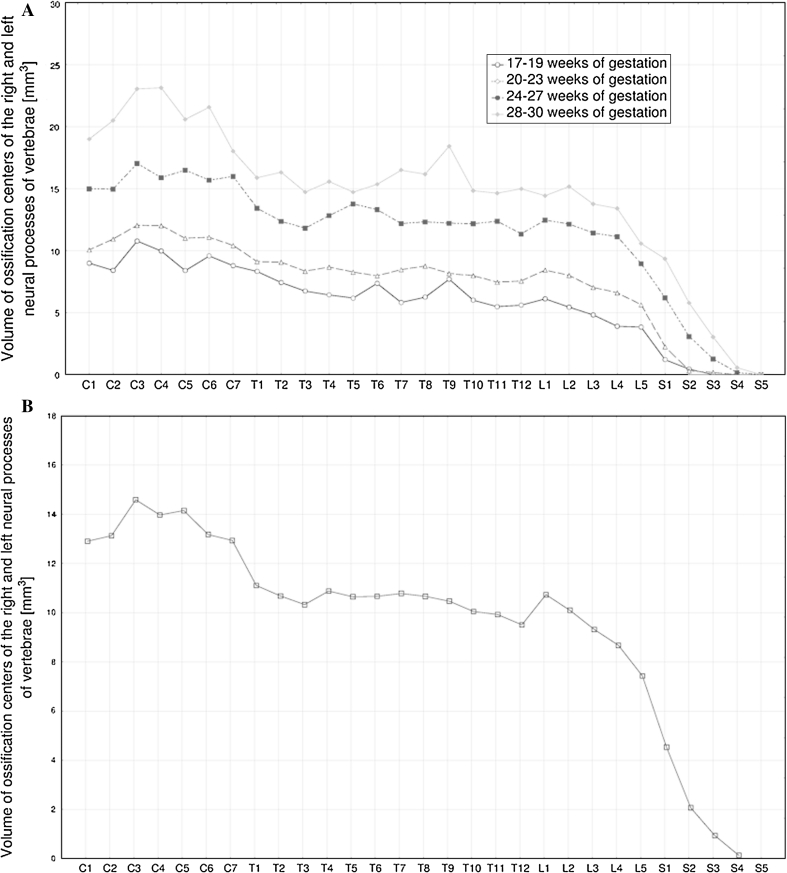
Mean volumes of neural ossification centers of the C1–S5 vertebrae in fetuses aged: 17–19, 20–23, 24–27, and 28–30 weeks of gestation (**a**), and for all fetuses (**b**)

The following four figures show the patterns for growth in length (Fig. 4), width (Fig. 5), cross-sectional area (Fig. 6), and volume (Fig. 7) of the neural ossification centers of vertebrae C1–S5 in fetuses aged 17–19, 20–23, 24–27 and 28–30 weeks. The growth dynamics for every parameter studied followed similarly in the four aforementioned age groups.

The mean values of the right and left neural ossification centers for the C1–S5 vertebrae have been presented in Table 2. Although there were insignificant laterality differences for the whole sample, the results were presented separately for the right and left sides, because of their considerable inter-individual variability.

**Table 2:** Morphometric parameters of the neural ossification centers of C1–S5 vertebrae

Vertebra	Neural ossification centers															
	Length (mm)				Width (mm)				Cross-sectional area (mm^2^)				Volume (mm^3^)			
	Right		Left		Right		Left		Right		Left		Right		Left	
	Mean	SD	Mean	SD	Mean	SD	Mean	SD	Mean	SD	Mean	SD	Mean	SD	Mean	SD
C1	5.82	1.29	5.36	1.55	2.14	0.65	1.98	0.69	10.78	4.51	10.30	3.87	13.08	4.73	12.85	4.88
C2	5.86	1.29	5.72	1.17	2.11	0.48	1.99	0.47	10.64	3.82	9.89	3.80	13.96	4.90	12.92	4.67
C3	5.78	1.55	5.83	1.42	2.26	0.70	2.25	0.88	11.57	4.11	10.56	4.60	15.03	4.96	14.36	5.16
C4	5.37	1.39	5.72	1.24	2.27	0.42	2.24	0.41	10.89	4.51	10.18	3.81	14.51	5.06	13.67	4.70
C5	5.75	1.44	5.88	1.56	2.11	0.48	2.00	0.49	10.46	4.17	9.97	4.17	14.14	5.07	13.86	5.94
C6	5.62	1.40	5.79	2.23	2.08	0.50	2.13	0.92	10.90	4.00	9.95	4.44	14.04	5.08	12.95	4.90
C7	5.42	1.49	5.27	1.62	2.28	1.08	2.19	0.54	9.51	3.54	9.59	3.69	12.82	5.03	12.70	4.75
T1	4.85	1.14	4.82	1.44	2.38	0.65	2.30	0.62	9.07	3.38	8.85	3.31	11.43	4.07	10.93	4.35
T2	5.01	1.14	4.94	1.04	2.24	0.58	2.20	0.67	9.03	3.29	8.32	3.06	10.97	3.73	10.52	3.77
T3	4.83	1.18	4.72	1.27	2.29	0.65	2.40	0.81	8.67	3.11	8.34	3.05	10.37	3.77	10.18	3.78
T4	4.77	1.16	4.90	1.17	2.14	0.64	2.39	0.69	8.68	3.27	8.84	3.37	10.64	4.31	10.69	4.35
T5	4.71	1.19	4.79	1.28	2.28	0.74	2.43	0.84	8.95	3.34	8.29	3.35	10.78	4.27	10.44	4.29
T6	4.60	1.18	4.64	1.21	2.08	0.58	2.22	0.56	8.24	2.96	8.36	3.47	10.43	4.05	10.47	4.51
T7	4.71	1.23	4.65	1.14	2.21	0.81	2.25	0.70	8.32	3.46	8.39	3.39	10.11	4.26	10.60	4.20
T8	4.64	1.17	4.52	1.18	2.08	0.72	2.12	0.60	8.73	3.65	8.21	3.38	10.72	4.33	10.45	4.63
T9	4.68	1.22	4.69	1.25	1.98	0.64	2.12	0.55	8.48	3.66	8.36	3.59	10.96	4.63	10.26	4.27
T10	4.47	1.26	4.53	1.27	1.97	0.63	1.97	0.63	7.85	3.20	7.80	3.37	10.23	4.06	9.82	4.41
T11	4.38	1.14	4.35	1.12	1.88	0.53	1.97	0.50	7.50	2.96	7.61	3.32	9.49	3.66	9.76	4.28
T12	4.58	1.14	4.54	1.10	1.77	0.42	1.80	0.42	7.40	3.27	7.62	2.68	9.49	3.96	9.37	3.45
L1	4.65	1.05	4.60	1.27	1.85	0.43	1.89	0.42	8.05	2.89	8.45	3.88	9.74	3.82	10.47	4.84
L2	4.52	1.25	4.64	1.27	1.95	0.60	1.88	0.50	8.10	3.64	8.09	3.73	9.70	4.32	9.97	4.48
L3	4.37	1.30	4.32	1.50	1.79	0.41	1.71	0.40	7.41	3.32	6.90	3.35	9.21	4.65	9.12	4.63
L4	4.07	1.22	4.23	1.34	1.69	0.45	1.67	0.46	6.50	3.51	6.45	3.29	8.37	4.69	8.55	4.66
L5	3.81	1.36	3.76	1.43	1.49	0.50	1.73	0.61	5.55	1.07	5.55	2.06	6.96	2.52	7.42	1.77
S1	2.61	0.94	2.65	0.98	1.03	0.70	1.06	0.75	3.43	1.37	3.46	1.48	4.44	2.01	4.37	1.46
S2	1.54	0.78	1.53	0.77	0.62	0.33	0.58	0.36	1.60	0.40	1.67	0.50	1.93	0.75	1.99	0.99
S3	0.75	0.40	0.77	0.43	0.30	0.26	0.30	0.17	0.70	0.23	0.69	0.38	0.87	0.36	0.90	0.30
S4	0.35	0.24	0.28	0.10	0.15	0.11	0.09	0.13	0.17	0.09	0.16	0.09	0.14	0.06	0.13	0.08
S5	0.00	0.00	0.00	0.00	0.00	0.00	0.00	0.00	0.00	0.00	0.00	0.00	0.00	0.00	0.00	0.00

The neural ossification centers were the longest ones in the cervical spine. The maximum values referred to the axis (5.86 ± 1.29 mm) on the right, and to C5 vertebra (5.88 ± 1.56 mm) on the left. The minimum values were related to C4 (5.37 ± 1.39 mm) and C7 (5.42 ± 1.49 mm) vertebrae on the right, and to C7 vertebra (5.27 ± 1.62 mm) on the left. There was a gradual decrease in length for neural ossification centers of T1–T12 vertebrae from 4.85 ± 1.14 to 4.58 ± 1.14 mm, and from 4.82 ± 1.44 to 4.54 ± 1.10 mm on the right and left sides, respectively. The length of lumbar neural ossification centers gradually decreased from the L1 vertebra on the right (4.65 ± 1.05 mm) and left (4.60 ± 1.27 mm) to L5 vertebra on the right (3.81 ± 1.36 mm) and left (3.76 ± 1.43 mm). Along the S1–S4 vertebrae, the neural ossification centers intensively declined from 2.61 ± 0.94 to 0.35 ± 0.24 mm, and from 2.65 ± 0.98 to 0.28 ± 0.10 mm on the right and left sides, respectively.

The neural ossification centers were the widest ones in the proximal thoracic spine, reaching the maximum values for T1 vertebra (2.38 ± 0.65 mm) on the right, and for T3–T5 vertebrae (2.40 ± 0.81 mm, 2.39 ± 0.69 and 2.43 ± 0.84 mm, respectively) on the left. Except for C3 (2.26 ± 0.70 and 2.25 ± 0.88 mm on the right and left, respectively) and C4 (2.27 ± 0.42 and 2.24 ± 0.41 mm on the right and left, respectively) vertebrae, a decrease in width of the neural ossification centers both in proximal and distal directions was found. As presented in Table 2, the widths of neural ossification centers of C1, C2 and C5 vertebrae were equivalent to that of T4, T6 and T8 vertebrae on the right, and to that of T10 and T11 vertebrae on the left. Furthermore, the widths of neural ossification centers of the following vertebrae: C3, C4, C7, T2, and T3 on the right attained similar values as did that of C3, C4, T1, T6, and T7 on the left. A decrease in width of neural ossification centers followed gradually in the distal thoracic-lumbar spine, and then conspicuously in the sacral spine, on the right and left sides respectively, from 2.08 ± 0.72 and 2.12 ± 0.60 mm for T8 vertebra through 1.49 ± 0.50 and 1.73 ± 0.61 mm for L5 vertebra, to 0.15 ± 0.11 and 0.09 ± 0.13 mm for S4 vertebra.

The growth dynamics for cross-sectional area were found to parallel that of volume of corresponding neural ossification centers. The largest cross-sectional areas of neural ossification centers were found in the first six cervical vertebrae, especially for C3 vertebra, reaching the value of 11.57 ± 4.11 mm^2^ on the right, and 10.56 ± 4.60 mm^2^ on the left. From there, the cross-sectional area started to decline, averaging on the right and left the following values: 9.51 ± 3.54 and 9.59 ± 3.69 mm^2^ for C7 vertebra, 8.24 ± 2.96 and 8.36 ± 3.47 mm^2^ for T6 vertebra, 8.05 ± 2.89 and 8.45 ± 3.88 mm^2^ for L1 vertebra, 3.43 ± 1.37 and 3.46 ± 1.48 mm^2^ for S1 vertebra, and 0.17 ± 0.09 and 0.16 ± 0.09 mm^2^ for S4 vertebra. Similarly, there was a gradual decrease in volume of the right and left neural ossification centers from the C3 vertebra (15.03 ± 4.96, 14.36 ± 5.16 mm^3^) through C7 (12.82 ± 5.03, 12.70 ± 4.75 mm^3^), T6 (10.43 ± 4.05, 10.47 ± 4.51 mm^3^), L1 (9.74 ± 3.82, 10.47 ± 4.84 mm^3^), and S1 (4.44 ± 2.01, 4.37 ± 1.46 mm^3^) vertebrae to S4 vertebra (0.14 ± 0.06, 0.13 ± 0.08 mm^3^).

## Discussion

The present examination describes the size of neural ossification centers throughout the fetal spine, providing the existing literature with completely novel quantitative data. The evidence material comprised eight results for each vertebra, thereby 232 results for each fetus, resulting in 12,760 individual numerical data for the whole sample. In our opinion, this study has been characterized by the following four advantages: a considerably numerous sample size (*n* = 55) without any detected malformations, a wide window width of obtained CT images from 1,404 to 1,692, precise and clearly defined parameters studied, and finally a reliable and objective method for measuring parameters in a direct manner, instead of deduced, extrapolated through a series of indirect measurements. The main disadvantage of this study may be a relatively narrow fetal age, ranging from 17 to 30 weeks of gestation. Another partial limitation may result from the fact that all measurements were performed by one observer in a blind fashion.

Our results did not support a significantly more rapid rate of ossification in females than in males, as it had previously been reported by Vignolo et al. [30]. Instead, in the material under examination there were insignificant differences in the size of the neural ossification centers of C1–S5 vertebrae.

In fact, the growth of neural ossification centers must follow three-dimensionally with a simultaneous increase in transverse and sagittal diameters, cross-sectional area, and volume. With relation to the neural ossifications centers of the C4 vertebra, Baumgart et al. [4] mathematically proved that on the right and left sides both their lengths (*y* = −19.601 + 8.018 × ln(Age) ± 0.369, *y* = −15.804 + 6.912 × ln(Age) ± 0.471) and widths (*y* = −5.806 + 2.587 × ln(Age) ± 0.146, *y* = −5.621 + 2.519 × ln(Age) ± 0.146) grew in a logarithmic fashion. Furthermore, both their cross-sectional areas (*y* = −9.188 + 0.856 × Age ± 2.174, *y* = −7.570 + 0.768 × Age ± 2.200) and volumes (*y* = −13.802 + 1.222 × Age ± 1.872, *y* = −11.038 + 1.061 × Age + 1.964) computed linear functions. Similarly, Szpinda et al. [25] found that the right and left neural ossification centers of the L3 vertebra increased logarithmically in length (*y* = −18.386 + 7.246 × ln (Age) ± 0.585, *y* = −23.171 + 8.766 × ln(Age) ± 0.753) and width (*y* = −5.014 + 2.176 × ln(Age) ± 0.218, *y* = −5.624 + 2.343 × ln(Age) ± 0.197), and proportionately in cross-sectional area (*y* = −12.122 + 0.847 × Age ± 1.351, *y* = −11.828 + 0.798 × Age ± 1.336) and volume (*y* = −10.902 + 0.854 × Age ± 2.141, *y* = −13.205 + 0.936 × Age + 1.603). To our knowledge, a much better understanding of evolving neural ossification centers may be gained by simultaneously studying them in the C1–S5 vertebrae in each specimen, as exemplified in the present study.

In any age-range, the length, CSA and volume of neural ossification centers decreased throughout the spine from proximal to distal, reaching the maximum values in the cervical spine, which were decreasing in a gradual manner in the thoracic-lumbar segment, and conspicuously in the sacral segment. The lengths of neural ossification centers of the axis on the right and C5 vertebra on the left were characterized by the largest values, 5.86 ± 1.29 and 5.88 ± 1.56 mm, respectively. The length of neural ossification centers of T1–T12–L1–L5 vertebrae decreased from 4.85 ± 1.14 through 4.58 ± 1.14 and 4.65 ± 1.05 mm to 3.81 ± 1.36 mm on the right, and from 4.82 ± 1.44 mm through 4.54 ± 1.10 and 4.60 ± 1.27 to 3.76 ± 1.43 mm on the left.

The right and left neural ossification centers of S1–S4 revealed an intensive decrease in length from 2.61 ± 0.94 to 0.35 ± 0.24 mm, and from 2.65 ± 0.98 to 0.28 ± 0.10 mm, respectively.

A gradual decrease in cross-sectional area of neural ossification centers followed from proximal to distal to display such a sequence for C3–C7–T6–L1–S1–S4 vertebrae: 11.57 ± 4.11, 9.51 ± 3.54, 8.24 ± 2.96, 8.05 ± 2.89, 3.43 ± 1.37, and 0.17 ± 0.09 mm^2^ on the right, and 10.56 ± 4.60, 9.59 ± 3.69, 8.36 ± 3.47, 8.45 ± 3.88, 3.46 ± 1.48, and 0.16 ± 0.09 mm^2^ on the left, respectively. A similar decreasing sequence concerning volumes of neural ossification centers of the aforementioned vertebrae followed as 15.03 ± 4.96, 12.82 ± 5.03, 10.43 ± 4.05, 9.74 ± 3.82, 4.44 ± 2.01, and 0.14 ± 0.06 mm^3^ on the right, and 14.36 ± 5.16, 12.70 ± 4.75, 10.47 ± 4.51, 10.47 ± 4.84, 4.37 ± 1.46, and 0.13 ± 0.08 mm^3^ on the left.

In turn, the width of neural ossification centers reached its maximum value in the proximal thoracic part, at the levels of T1 vertebra (2.38 ± 0.65 mm) on the right and T3–T5 (2.40 ± 0.81, 2.39 ± 0.69, and 2.43 ± 0.84 mm, respectively) vertebrae on the left, to decline in both proximal and distal directions. Therefore, the widths of neural ossification centers of proximal cervical vertebrae (C1, C2, and C5) were equivalent to that of T4, T6, and T8 thoracic vertebrae on the right, and T10 and T11 vertebrae on the left. The width of neural ossification centers decreased gradually in the distal thoracic-lumbar spine, and intensively in the sacral spine, for the right and left T8–L5–S4 vertebrae from 2.08 ± 0.72 mm through 1.49 ± 0.50 to 0.15 ± 0.11 mm, and from 2.12 ± 0.60 mm through 1.73 ± 0.61 to 0.09 ± 0.13 mm, respectively.

As presented above, the largest values for lengths, cross-sectional areas and volumes of neural ossification centers referred to the cervical vertebrae. In our opinion, this is a direct consequence of the timing of ossification, since the neural processes ossify in a predictable pattern, probably starting with the cervical part of the spine [2, 3, 19]. The present results have supported the fact that the ossification sequence of neural processes progresses from cervical to sacral. Such a predominant increase in size of neural ossification centers in the cervical spine found in the material under examination may result from the fact that neural processes comprise an extensive area for anchorage of nuchal muscles [2], being responsible for early head extension in the human fetus, the so called “gasp reflex”. On the other hand, with relation to the ossification centers of C1–S5 vertebral bodies [22] there was a gradual increase in all values from the axis until T5 vertebra for the sagittal diameter, until T12 vertebra for the transverse diameter, until L2 vertebra for the cross-sectional area, and finally until L3 vertebra for the volume, which supports the start of the body ossification centers with the inferior thoracic-superior lumbar spine, and then its simultaneous cranial and caudal progression [2, 3, 19].

To our knowledge, a sharp decrease in all values of the sacral segment is the consequence of a delayed appearance of sacral ossification centers. As reported by de Biasio et al. [5], in fetuses at ages of 17 weeks, the body ossification centers in the sacral spine were visible in all fetuses for S1 and S2, in 75 % for S3, in 12.5 % for S4, and in no fetus for S5. In the material under examination, the neural ossification centers of the sacral segment occurred in 41 (74.5 %) fetuses for S1, 34 (61.8 %) fetuses for S2, 29 (52.7 %) fetuses for S3, 7 (12.7 %) fetuses for S4, and in no fetus for S5.

The ossification progress within neural processes is relevant in the ultrasound diagnosis and monitoring of neural tube defects [4, 8, 10, 13–16]. Hence, many spinal abnormalities including achondrogenesis, caudal regression syndrome, diastematomyelia, and skeletodysplasias may ultrasonografically be diagnosed and monitored in the fetus [26, 28, 30]. The sacral bodies were reported to ossify earlier than the sacral arches [5]. Thus, delayed ossification of the sacral bodies with relation to the sacral arches occurs in achondrogenesis [5, 26, 28]. Caudal regression syndrome ranges from isolated sacral agenesis to the lack of the lumbosacral spine [5]. A sagittal cleft of the spinal cord (a spinal dysraphism) with splaying of vertebral arches is typical of diastematomyelia [17, 27, 28]. The most conspicuous type of spina bifida is undoubtedly myelomeningocele with the unfused neural processes of the lumbosacral spine. As a result, the spinal cord freely protrudes through an existing opening [7, 11, 15]. In fact, an understanding of the timing of spinal ossification centers is extremely useful in the prenatal detection of skeletal dysplasias (osteochondrodysplasias) resulting in both a delayed appearance of ossification centers and poor mineralization because of osteogenesis imperfecta type II [28], achondrogenesis [26], tanatophoric dysplasia type I [11], and hypophosphatasia [31].

In summary, the present study is the first in the medical literature to provide objective information on the quantitative growth of the neural ossification centers throughout the fetal spine, and simultaneously emphasizes its clinical aspects.

## Conclusions

1. The neural ossification centers show neither male–female nor right–left differences.
2. The neural ossification centers are characterized by the maximum length for C2–C6 vertebrae, the maximum width for the proximal thoracic spine, and both the maximum cross-sectional area and volume for C3 vertebra.
3. There is a sharp decrease in size of the neural ossification centers along the sacral spine.
4. A decreasing sequence of values for neural ossification centers along the spine from cervical to sacral appears to parallel the same direction of the timing of ossification.
5. The quantitative growth of the neural ossification centers is of potential relevance in the prenatal diagnosis and monitoring of achondrogenesis, caudal regression syndrome, diastematomyelia and spina bifida.
